# An Ideal Molecular Construction Strategy for Ultra‐Narrow‐Band Deep‐Blue Emitters: Balancing Bathochromic‐Shift Emission, Spectral Narrowing, and Aggregation Suppression

**DOI:** 10.1002/advs.202307675

**Published:** 2023-12-31

**Authors:** Xiaofeng Luo, Qian Jin, Mingxu Du, Dong Wang, Lian Duan, Yuewei Zhang

**Affiliations:** ^1^ Key Lab of Organic Optoelectronics and Molecular Engineering of Ministry of Education Department of Chemistry Tsinghua University Beijing 100084 P. R. China; ^2^ Laboratory of Flexible Electronics Technology Tsinghua University Beijing 100084 P. R. China; ^3^ Applied Mechanics Lab School of Aerospace Engineering Tsinghua University Beijing 100084 P. R. China

**Keywords:** decoration strategy, deep‐blue OLEDs, high efficiency and stability, multiple resonance, ultra‐narrow‐band

## Abstract

Narrowband emissive multiple resonance (MR) emitters promise high efficiency and stability in deep‐blue organic light‐emitting diodes (OLEDs). However, the construction of ideal ultra‐narrow‐band deep‐blue MR emitters still faces formidable challenges, especially in balancing bathochromic‐shift emission, spectral narrowing, and aggregation suppression. Here, DICz is chosen, which possesses the smallest full‐width‐at‐half‐maximum (FWHM) in MR structures, as the core and solved the above issue by tuning its peripheral substitution sites. The 1‐substituted molecule Cz‐DICz is able to show a bright deep‐blue emission with a peak at 457 nm, an extremely small FWHM of 14 nm, and a CIE coordinate of (0.14, 0.08) in solution. The corresponding OLEDs exhibit high maximum external quantum efficiencies of 22.1%–25.6% and identical small FWHMs of 18 nm over the practical mass‐production concentration range (1–4 wt.%). To the best of the knowledge, 14 and 18 nm are currently the smallest FWHM values for deep‐blue MR emitters with similar emission maxima under photoluminescence and electroluminescence conditions, respectively. These discoveries will help drive the development of high‐performance narrowband deep‐blue emitters and bring about a revolution in OLED industry.

## Graphical abstract

Toward the goal of ideal ultra‐narrow‐band deep‐blue emitters, a novel “decoration strategy” is proposed to balance the bathochromic‐shift emission, spectral narrowing, and aggregation suppression. The optimized OLEDs exhibit high maximum external quantum efficiencies of 22.1%−25.6% with similar emissions at ≈461 nm and identical small full‐width‐at‐half‐maxima of 18 nm in the practical mass‐production concentration range (1–4 wt.%).

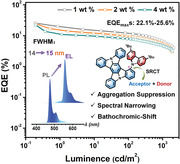

## 1 Introduction

As a new generation of fantastic displays, organic light‐emitting diodes (OLEDs) have been widely used in many scenarios of life with excellent characteristics such as flexibility, power saving, and design versatility.^[^ ^1^ ^]^ Currently, the pixels of organic light‐emitting diodes (OLEDs) are arranged by red, green, and deep‐blue emitters in a certain ratio. Among them, the efficiency and stability of green/red phosphorescent materials have fulfilled the mass production requirements,^[^ ^2^ ^]^ while the device performances of deep‐blue dopants are still far from satisfactory.^[^ ^3^ ^]^ On one hand, conventional fluorescent dyes, although they can produce stable electroluminescences (ELs), are inefficient in their devices (unable to utilize the non‐radiating triplet (T_1_) excitons).^[^ ^1d^ ^]^ On the other hand, while conventional phosphors^[^ ^4a^ ^]^ and thermally activated delayed fluorescence (TADF)^[^ ^4b^ ^]^ dopants can effectively recycle T_1_ excitons, the onset of the emission wavelengths are usually shorter than 400 nm (corresponding to energies higher than 3.10 eV, which is a very high excitation energy that greatly increases the possibility of molecular degradation) and the exciton decay processes are pretty slow, both of which can impair device stability.^[^ ^5^ ^]^ Thus, the realization of stable deep‐blue OLEDs faces formidable challenges and needs new conceptual advancements in molecular design.

**Figure 1:**
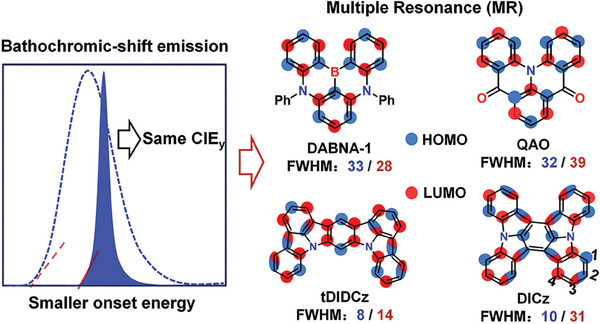
The relationship between the CIE*_y_* /onset energy with FWHM for deep‐blue OLEDs (left) and representative narrowband‐emissive MR‐ skeletons. The blue and red numbers represent the FWHM values at photoluminescence and electroluminescence conditions, respectively.

Recently, multiple resonance (MR) materials have attracted tremendous attention by utilizing the different resonance effects of their donor (D) and acceptor (A) atoms to achieve a special separation of frontier molecular orbitals (FMOs) at the atomic level, thus greatly suppressing the structural relaxation and vibrational coupling between the ground and excited states to obtain the narrowband emissions.^[^ ^6^, ^7^, ^8^, ^9^ ^]^ It is noteworthy that similar CIEy values can be reached with relatively redshifted emission peaks using these narrowband emitters as compared to ones with larger full width at half maxima (FWHMs). This gives us some new technological insights in high‐performance deep‐blue OLEDs, namely that significantly reduced peaking and onset energies can be realized by constructing narrowband emissive deep‐blue materials, which implies much lower excited‐state energies and thus naturally contributing to long‐term stabilities (**Figure** **1**). Considering that the narrower the emission band, the smaller the CIEy value can be realized for a same emission peak, the construction of ultra‐narrow‐band emitters with longer wavelengths (about 460 nm)^[^ ^5c^ ^]^ is an effective way to solve the difficulties in practical realization of deep‐blue OLEDs.

Exhilaratingly, indolo[3,2,1‐jk]carbazole (ICz) based MR system brings the design concept of ultra‐narrow‐band emitters,^[^ ^8^, ^9^ ^]^ which makes it easier to obtain smaller FWHM values in deep‐blue region compared to other MR systems (B/N^[^ ^6^ ^]^ and N/C = O,^[^ ^7^ ^]^ Figure 1). Although the smallest intrinsic FWHMs of 8 (tDIDCz, peaking at 401 nm)^[^ ^8a,h^ ^]^ and 10 nm (DICz, peaking at 446 nm)^[^ ^8b‐d^ ^]^ can be achieved in the violet and deep‐blue regions, the corresponding EL spectra are significantly broadened to 14 nm (tDIDCz) and 31 nm (DICz) for their large planar molecular structures. Attempts have been made to suppress the negative effects of concentration, but it remains challenging to find a strategy that balances the bathochromic‐shift emission and ultra‐narrow spectrum. For example, the introduction of peripheral neutral site‐blocking groups, while maintaining ultra‐small FWHMs and suppressing concentration aggregation, has virtually no effect on wavelengths;^[^ ^8b,e^ ^]^ and the introduction of peripheral electron‐donating/absorbing groups, while enabling bathochromic‐shifted emissions, sacrifices color purity considerably.^[^ ^8h,i^ ^]^ In addition, the introduction of peripheral groups at different positions is also difficult to synthesize.^[^ ^8f,j^ ^]^ Therefore, there is an urgent need for more advanced molecular design strategies to address the above issues.

Herein, DICz with an emission wavelength of 446 nm was chosen as the MR core, and by tuning its peripheral substitution sites, an ideal molecular construction strategy for ultra‐narrow‐band deep‐blue emitters that encompasses bathochromic‐shift emission, spectral narrowing, and aggregation suppression was proposed. On one hand, in order to achieve the “steric wrapping” effect, the peripheral substituents need to have a large spatial resistance to the central MR skeleton, and thus the substituent positions are preferably 1 and 4 (Figure 1). On the other hand, it was previously reported that indolo[3,2,1‐jk]carbazole (ICz) can be used as an acceptor unit for conventional TADF emitters,^[^ ^10^ ^]^ and thus it can be inferred that the construction of a short‐range charge‐transfer state (SR‐CT) by introducing a suitable donor at position 1 or 4 of DICz can simultaneously achieve an effective spectral red‐shift and maintain an ultra‐small FWHM value. Considering that the introduction of a peripheral donor at position 4 can produce a larger S_0_ (ground‐state)‐S_1_ (excited‐state) structural deformation, the decoration strategy in 1‐position of DICz is a more preferred approach to construct ideal ultra‐narrow deep‐blue MR emitters. As envisioned, the proof‐of‐concept emitter Cz‐DICz shows a bright deep‐blue emission with a peak at 457 nm, an extremely small FWHM of 14 nm, and a CIE coordinate of (0.14, 0.010). Benefiting from the high photoluminescence quantum yields (PLQYs > 96%) and suppressed chromophore interactions, maximum external quantum efficiencies (EQE_max_s) up to 22.1%−25.6% and identical small FWHMs of 18 nm were simultaneously achieved in the practical mass‐production concentration range (1–4 wt.%). To the best of our knowledge, 14 and 18 nm are currently the smallest FWHM values for deep‐blue MR emitters (peaking ≈460 nm) under PL and EL conditions, respectively, validating the effectiveness of this molecular design strategy.

## 2 Results and Discussion

**Figure 2:**
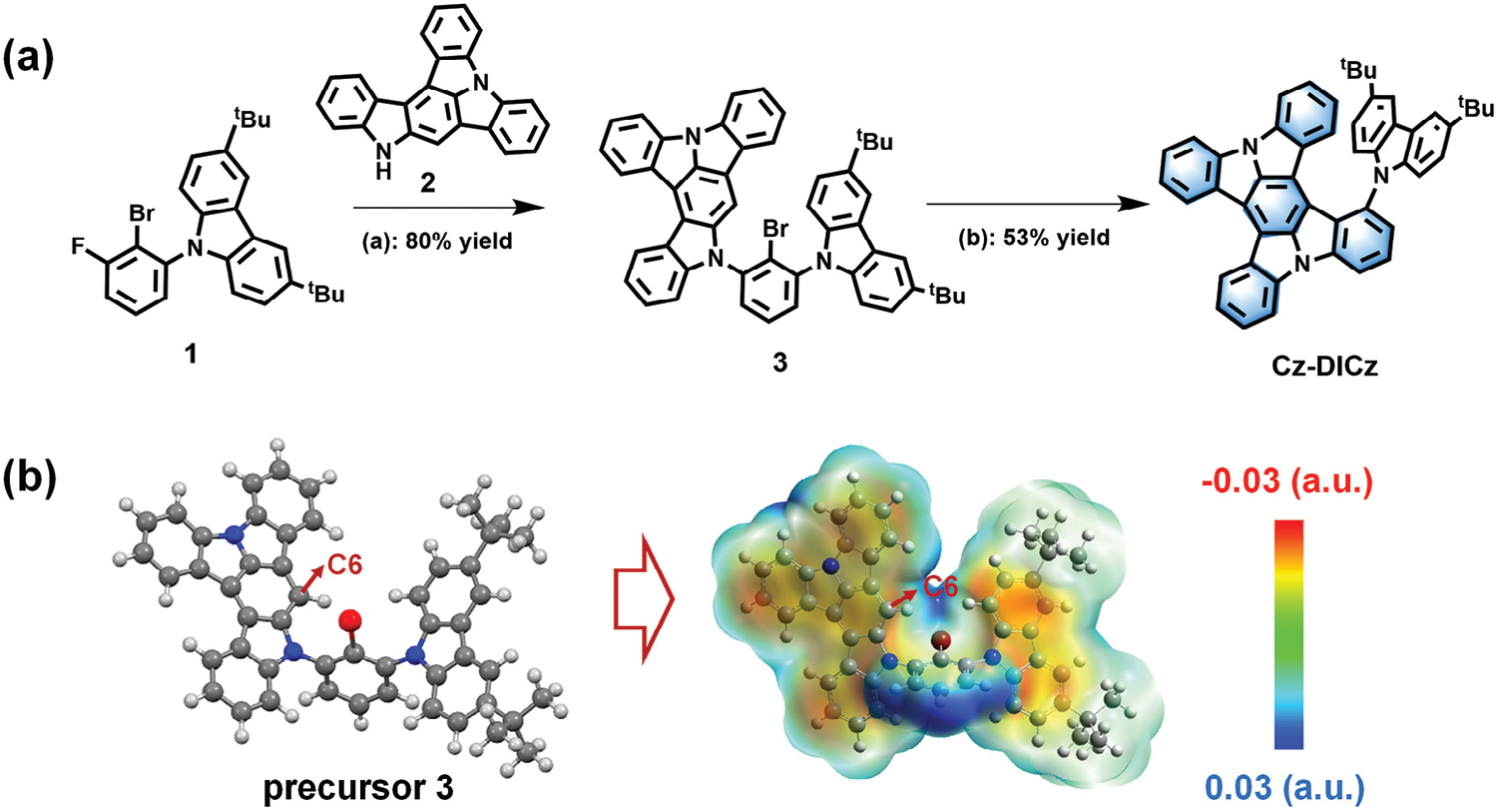
a) The synthetic procedures of Cz‐DICz: i) Cs_2_CO_3_, DMF, reflux, 12 h; ii) Pd(OAc)_2_, Bu_4_N^+^Br^−^, PPh_3_, K_2_CO_3_, DMAC, reflux, 12 h. b) The optimized structure of precursor 3 and its electrostatic potential iso‐surface with minimum point.

The synthesis procedure of Cz‐DICz is illustrated in **Figure** **2**a and Scheme S1 (Supporting Information). Starting from the reported compounds 1 and 2,^[^ ^11^ ^]^ the key precursor 3 could be efficiently prepared by the Buchwald‐Hartwig coupling method. Benefiting from the fact that the carbon atom (C6) of precursor 3 overlaps with a minimal electrostatic potential (ESP) point (Figure 2a), we successfully carried out a highly selective electrophilic C‐C cyclization reaction using a conventional method to obtain the target molecule Cz‐DICz with up to 53% yield. Cz‐DICz exhibited adequate gas, moisture, heat resistance and was fully characterized by NMR spectroscopy, mass spectrometry, and elemental analysis.

**Figure 3:**
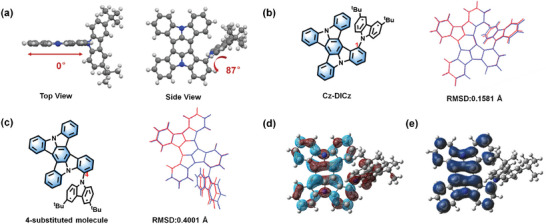
a) Crystal structure of Cz‐DICz. b) The molecular structure of 1‐substituted molecule (Cz‐DICz), S_0_ (blue)‐S_1_ (red) structural deformation and corresponding RMSD value. c) The molecular structure of 4‐substituted molecule, S_0_ (blue)‐S_1_ (red) structural deformation and corresponding RMSD. d) The distributions of the HOMO (wine color) and LUMO (cyan color) of Cz‐DICz. e) The triplet spin density distribution of Cz‐DICz.

Cz‐DICz single crystals were collected by slow evaporation in a tetrahydrofuran/methanol (1:3) solution to further confirm the molecular structure. As depicted in **Figure** **3**a, the torsion angle between the peripheral donor and DICz skeleton is as large as 87°, which not only provided a large spatial resistance to inhibit concentration aggregation, but also facilitated the acquisition of SRCT. To verify the superiority of the above molecular design, we further performed density functional theory (DFT) and time‐dependent DFT (TD‐DFT) calculations. Since spectral broadening is related to the structural displacement between S_1_ and S_0_,^[^ ^6d^ ^]^ we first calculated the root‐mean‐square displacements (RMSDs) of the 1 and 4‐substituted compounds in order to compare the spectral narrowing ability of these two positions. As shown in Figure 3b,c, the target molecule Cz‐DICz has a smaller RMSD value (0.1581 Å, 1‐substituted product) compared to the 4‐substituted one (0.4001 Å), predicting a smaller FWHM value. Thus, the decoration strategy in 1‐position of DICz is a more efficient approach to construct the ideal ultra‐narrow‐band deep‐blue MR emitters. For Cz‐DICz, the most notable feature was that the introduction of carbazole unit led to an expansion of HOMO onto the peripheral donor, while LUMO remained on the DICz core (Figure 3d). That is, the peripheral donor unit enhanced the intramolecular charge transfer (ICT) property of Cz‐DICz, resulting in a narrower energy gap (*E*_g_) value and thus a red‐shifted emission (Figure S1, Supporting Information). Considering the nearly orthogonal structural feature of the peripheral donor and DICz skeleton, i.e., a limited influence on the MR effect of DICz core, it is fair to assume that the SRCT property and ultra‐narrow‐band emission can be maintained. The above results were also well supported by natural transition orbitals (NTOs) for the S_1_ and T_1_ excitations (Figure S2 and Table S1, Supporting Information). Furthermore, considering that the triplet annihilations have been proven to cause the exciton annihilations, the spin density distribution (SDD) of the lowest excited triplet state of Cz‐DICz was also calculated (Figure 3e). As expected, the triplet state was confined to the DICz core, suggesting that the bulk Cz unit can also act as an electronically inert moiety to introduce a large steric effect, thus solving the problem raised by dopant aggregation.

**Figure 4:**
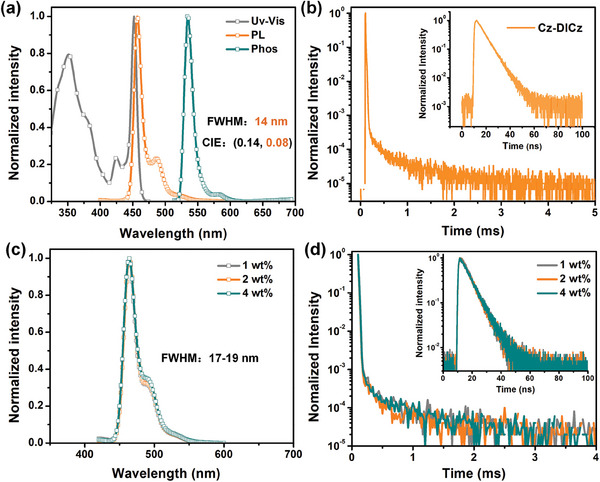
a) Ultraviolet/visible (UV/Vis) absorption, fluorescence (298 K), and phosphorescence (77 K) spectra of Cz‐DICz in toluene (10^−5^ m). b) Transient PL decay curves of Cz‐DICz in toluene after N_2_‐bubbling for 10 min. Inset: prompt PL decay in the nanosecond range. c) The emission spectra of the doped films with different dopant concentrations in mCBP: x wt.% Cz‐DICz (*x* = 1, 2, 4). d) Transient PL decay curves of the doped films with different dopant concentrations in mCBP: x wt.% Cz‐DICz (*x* = 1, 2, 4). Inset: prompt PL decays in the nanosecond range.

The photophysical proprieties of Cz‐DICz, including the UV/Vis absorption, fluorescence and phosphorescence spectra, were initially studied with dilute toluene solutions (**Figure** **4**), and the corresponding data are presented in **Table** **1**. Cz‐DICz showed a sharp absorption band at wavelength of 452 nm, corresponding to the unique short‐range charge transfer (SR‐CT) transition (Figure 4a). The absorption bands below 410 nm were attributed to the π‐π* transitions of the rigid PAHs, while the absorption band at ≈425 nm was assigned to the n‐π* transition. The photoluminescence (PL) spectrum depicted a mirror image compared to the absorption spectrum with a maximum at 457 nm, an FWHM of 14 nm, and CIE coordinates of (0.14, 0.08). To the best of our knowledge, 14 nm is currently the smallest FWHM value for the practical deep‐blue MR emitters (peaking around 460 nm) under PL conditions (Table S2, Supporting Information).^[^ ^7^, ^8^, ^9^ ^]^ Combining the absorption and PL peaks, an extremely small Stokes shift of 5 nm was obtained, indicating the negligible S_1_‐S_0_ structural displacement. When the solvent polarity was changed from a weak (n‐hexane) to a strong (dichloromethane) one, the bathochromic shift of the emission spectrum is merely 5 nm, evidencing the SRCT characteristics of the singlet (Figure S7, Supporting Information). It is noteworthy that in n‐hexane, the FWHM value of Cz‐DICz is as small as 12 nm, which is almost identical to the parent nucleus of DICz, indicating the advantage of the above molecular design in balancing the spectral redshift and broadening. The phosphorescence spectrum of Cz‐DICz was also measured in frozen dilute toluene at 77 K. The calculated S_1_ and T_1_ energies based on the peak of the fluorescence and onset of phosphorescence spectra were 2.71 and 2.37 eV, respectively, resulting in an S_1_‐T_1_ energy difference (Δ*E*_ST_) of 0.34 eV, which is sufficient for reverse intersystem crossing (RISC) under ambient conditions. As shown in Figure 4b, the PL decay curve of Cz‐DICz exhibited a clear second‐order exponential decay, corresponding to prompt (*τ*_PF_: 5.5 ns) and delayed (*τ*_DF_: 405 µs) fluorescence, respectively. After incorporating the high PLQY value of 98.6%, the calculated rate constants for radiative decay (*k*_r_) and RISC (*k*_RISC_) are 1.8 × 10^8^ s^−1^ and 2.6 × 10^3^ s^−1^, respectively.^[^ ^12^ ^]^ Compared to conventional TADF emitters with long‐range charge‐transfer state (LR‐CT) properties, Cz‐DICz has a significantly longer TADF lifetime and smaller *k*_RISC_ rate, which may be related to the smaller recombination energy in the Marcus‐Levich‐Jortner theory.^[^ ^13^ ^]^

**Table 1:** Summary of the photophysical data of Cz‐DICz.

	S_1_ ^a)^ [eV]	T_1_ ^a)^ [eV]	ΔE_ST_ ^a)^ [eV]	λ_abs_ ^a)^ [nm]	λ_PL_ [nm]	FWHM [nm]	CIEy^a)^	PLQY [%]	*k*_r_ ^a)^ [10^8^ s^−1^]	HOMO [eV]	LUMO [eV]
Cz‐DICz	2.71	2.37	0.34	452	457^a)^/460^b)^	14^a)^/17^b)^	0.08	98.6^a)^/98.4^b)^	1.8	−5.55^c)^	−2.59^d)^

^a)^ Measured in the toluene solution with a concentration of 10^−5^ mol L^−1^ (N_2_);

^b)^ Measured in 1.0 wt.% doped film;

^c)^ Measured in dry dichloromethane with a concentration of 10^−3^ mol L^−1^;

^d)^ Measured in dry *N,N*‐dimethylformamide with a concentration of 10^−3^ mol L^−1^.

The photophysical properties of Cz‐DICz were further investigated by dispersing it in a wide‐energy‐gap host, 3,3′‐di(carbazol‐9‐yl)biphenyl (mCBP), with the doping concentrations ranging from 1 wt% to 4 wt%. As shown in Figure 4c, unlike the behaviors of conventional MR emitters, the charge transfer (CT)‐induced bathochromic‐shift emission, spectral broadening, and concentration quenching are well balanced in Cz‐DICz films, which consistently have similar emission maxima (λ: 464 nm), small FWHMs (17–19 nm), and high PLQYs (96.6%−98.4%) over the practical mass‐production concentration range (1–4 wt.%). The relatively broader spectra of Cz‐DICz in doped films could be assigned to the π–π interaction between the dopant and host material as well as the conformational change, which is a common phenomenon in MR systems.^[^ ^14^ ^]^ Temperature‐dependent transient PL decay test confirmed the TADF nature of Cz‐DICz,^[^ ^15^ ^]^ with a short‐lived transient lifetime of 5.9 ns and a long‐lived delayed lifetime of 426 µs in 1 wt.% doped film at room temperature (Figure S8, Supporting Information). Notably, the lifetimes of the delayed components remain essentially constant with increasing the doping concentration, further suggesting that there is no significant molecular aggregation in this concentration range (Figure 4d). Given the excellent photophysical properties of Cz‐DICz, we believe that the 1‐position decoration strategy of DICz is an efficient approch to obtain the ideal narrowband deep‐blue MR emitters.

**Figure 5:**
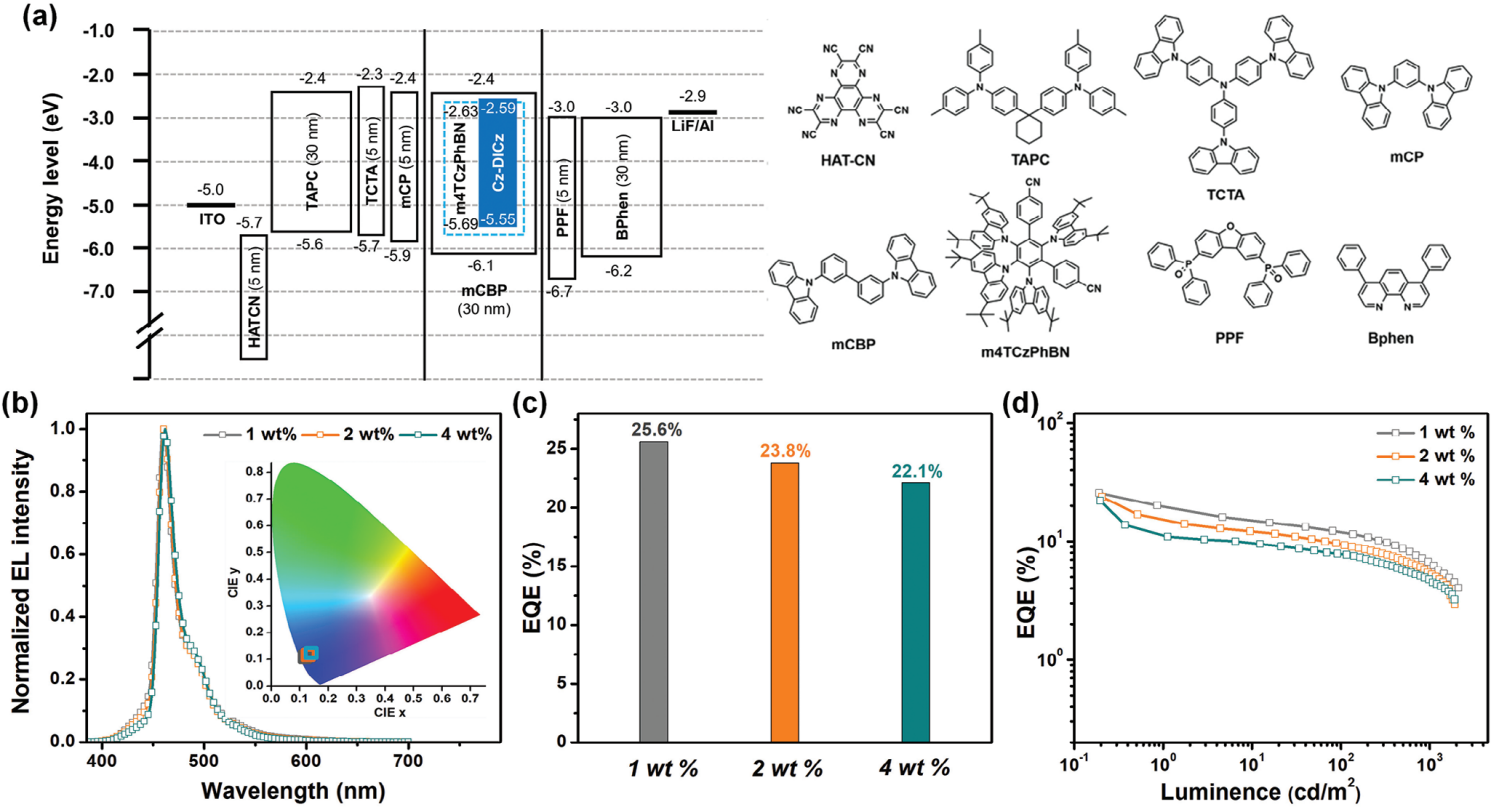
a) Device configuration, energy level, and molecular structure of the materials used. b) The EL spectra and CIE color coordinates at 10 mA cm^−2^. c) The *EQE*_max_ values versus doping concentrations. d) External quantum efficiency versus luminance characteristics.

Owing to the excellent thermal stability with high decomposition (*T*_d_ : 466°C) temperature (Figure S9, Supporting Information), OLEDs with the structure of indium‐tin‐oxide (ITO, 135 nm)/HATCN (1,4,5,8,9,11‐Hexaazatriphenylene hexacarbonitrile, 10 nm)/TAPC (1,1‐bis[4‐[*N*,*N′*‐di(*p*‐tolyl)amino]phenyl]‐cyclohexane, 30 nm)/TCTA (4,4′,4′‐ tris(carbazol‐9‐yl)‐triphenylamine, 5 nm)/mCP (1,3‐di‐9‐carbazolyl‐benzene, 5 nm)/mCBP: 30 wt.% (2′s,4′r,5′r,6′s)−2′,4′,5′,6′‐tetrakis(3,6‐di‐*tert*‐butyl‐9H‐carbazol‐9‐yl)‐[1,1′,3′,1″‐terphenyl]−4,4″‐dicarbonitrile (m4TCzPhBN): 1, 2, 4 wt.% DBCz‐Mes (30 nm)/PPF (2,8‐bis(diphenylphosphoryl)‐dibenzo[b,d]furan, 5 nm)/Bphen (4,7‐diphenyl‐1,10‐phenanthroline, 30 nm)/LiF (0.5 nm)/Al (150 nm) were fabricated to evaluate the potential of Cz‐DICz as a deep‐blue emitter. Among them, HATCN and LiF were used as hole‐ and electron injection layers; TAPC and Bphen were served as hole and electron transport layers; TCTA/mCP and PPF were chosen as electron‐ and hole‐blocking layers. The energy level diagram of the devices as well as the molecular structures of the materials adopted in organic layers are shown in **Figure** **5**a. Considering the slow RISC rate of Cz‐DICz, we employed a TADF sensitizer to accelerate the harvest of triplet excitons, which was a conventional strategy in MR‐OLEDs (please see Supporting Information for the non‐sensitized device performances). Herein, m4TCzPhBN was selected as the sensitizer for its high PLQY (90%), fast *k*_RISC_ (≈1.0 × 10^6^ s^−1^), and a significant overlap with the absorption spectrum of Cz‐DICz for efficient Förster energy transfer (FET, Figure S11, Supporting Information).^[^ ^16^ ^]^ And the radius (R_0_) of the FET (defined as the intermolecular distance at which the energy transfer rate constant is equal to the total decay rate constant of the pristine donor without acceptor) was calculated to be as large as 3.64 Å.^[^ ^17^ ^]^ Figures S11 and S12 and Table S4 (Supporting Information) provide a detailed explanation of the mechanism of TADF‐sensitized fluorescence emission and the associated photophysical processes.

As illustrated in Figure 5b and Table S5 (Supporting Information), all devices emitted similar deep‐blue emissions at around 460 nm with an identical small FWHM of 18 nm, which was consistent with the PL results for the doped films. To the best of our knowledge, 18 nm represented one of the narrowest EL spectra (irrespective of color) in the literature (Table S2, Supporting Information),^[^ ^7^, ^8^, ^9^ ^]^ validating the effectiveness of the molecular design strategy described above in balancing the bathochromic‐shift emission, spectral narrowing, and aggregation suppression. Notably, the corresponding CIE coordinates ranged from (0.14, 0.10) to (0.14, 0.11), which were approaching blue point (0.14, 0.08) defined by the National Television System Committee. The EQE_max_‐dopant concentration relationships are shown in Figure 5c,d. High EQE_max_s of 22.1%−25.6% could be could be achieved for for the above OLEDs, which were among the best results of previously reported narrowband deep‐blue OLEDs based on ICz‐MR emitters (Figure 5c,d).^[^ ^8^ ^]^ Due to the lack of high‐performance deep‐blue sensitizers, the EQE values at high brightness were not ideal, which could be further improved by developing TADF sensitizers with superior properties (e.g., higher PLQY and *k*_RISC_ values).

**Figure 6:**
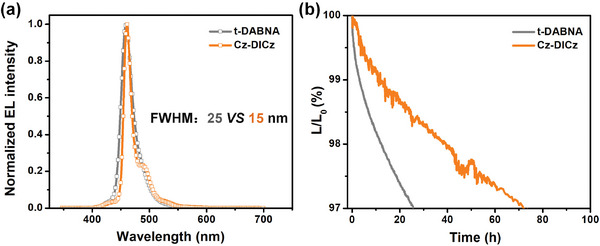
a) EL spectra recorded at 10 mA cm^−2^. b) Lifetime data at a constant current density of 15 mA cm^−2^ of the fluorescent OLEDs based on Cz‐DICz and t‐DABNA.

Besides efficiency, device lifetime is also a parameter of great interest. Considering that commercial blue OLEDs are based on the triplet‐triplet annihilation (TTA) mechanism, anthracene derivative sensitized device was further constructed to evaluate the intrinsic stability of Cz‐DICz. The well known stable narrowband deep‐blue emitter *t*‐DABNA was chosen as a comparison.^[^ ^18^ ^]^ As shown in **Figure** **6**a, S18, and Table S6 (Supporting Information), compared with *t*‐DABNA (FWHM = 25 nm and EQE_max_ = 9.0%), the Cz‐DICz device shows not only a significantly narrower EL (FWHM = 15 nm) but also a much higher EQE_max_ (9.4%) at a similar ≈460 nm emission wavelength. Impressively, at a constant current density of 15 mA cm^−2^, the Cz‐DICz device showed an excellent LT97 (time to lose 3% of the initial luminance) of 72.5 h, which was 2.8 times that of the control device based on *t‐*DABNA (25.8 h) and one of the best performances available for deep‐blue OLEDs (Figure 6b).^[^ ^9^, ^19^ ^]^ The excellent device performance described above further demonstrates that the decoration strategy in 1‐position of DICz is an effective method for constructing ideal utral‐narrow deep‐blue MR emitters.

## 3 Conclusion

In summary, toward the goal of ideal ultra‐narrow‐band deep‐blue emitters, a novel “decoration strategy” was proposed to balance the bathochromic‐shift emission, spectral narrowing, and aggregation suppression. The proof‐of‐concept emitter Cz‐DICz (1‐substituted molecule) is capable of displaying ultrapure deep‐blue emission (CIEy: 0.08) with a peak at 457 nm, an extremely small FWHM of 14 nm, and a high PLQY of 98.6% in solution. To the best of our knowledge, 14 nm represents the smallest FWHM value for deep‐blue MR emitters with similar emission maximum. The optimized OLED devices exhibit high EQE_max_s of 22.1%−25.6% with similar emissions at around 461 nm and identical small FWHMs of 18 nm in the practical mass‐production concentration range (1–4 wt.%). Notably, benefiting from the ultra‐small FWHM (15 nm) and proper peak position (461 nm, S_1_ energy down to 2.69 eV), the TTA device shows an excellent LT97 of 72.5 h, which is one of the best performances for deep‐blue OLEDs. We believe this strategy will continue to drive the development of high‐performance deep‐blue OLEDs.

## Conflict of Interest

The authors declare no conflict of interest.

## Supporting information

Supporting Information

## Data Availability

The data that support the findings of this study are available from the corresponding author upon reasonable request.
