# Supplementary material for: An Ideal Molecular Construction Strategy for Ultra‐Narrow‐Band Deep‐Blue Emitters: Balancing Bathochromic‐Shift Emission, Spectral Narrowing, and Aggregation Suppression
Source: Adv Sci (Weinh). 2023 Dec 31;11(11):2307675. doi: 10.1002/advs.202307675 (PMC10953554; doi:10.1002/advs.202307675)
Supplement: Supplementary file 1 — Supporting Information [file ADVS-11-2307675-s001.pdf]

ADVANCED SCIENCE
Open Access

Supporting Information

for *Adv. Sci.*, DOI 10.1002/advs.202307675

An Ideal Molecular Construction Strategy for Ultra-Narrow-Band Deep-Blue Emitters: Balancing Bathochromic-Shift Emission, Spectral Narrowing, and Aggregation Suppression

*Xiaofeng Luo, Qian Jin, Mingxu Du, Dong Wang, Lian Duan and Yuewei Zhang**

Supporting Information

An Ideal Molecular Construction Strategy for Ultra-Narrow-Band Deep-Blue Emitters: Balancing Bathochromic-Shift Emission, Spectral Narrowing, and Aggregation Suppression

*Xiaofeng Luo, Qian Jin, Mingxu Du, Dong Wang, Lian Duan, and Yuewei Zhang**

General Information. All commercially available reagents were used as received unless otherwise stated. All reactions were carried out using Schlenk techniques under a nitrogen atmosphere. 600 MHz ^{1}H-NMR spectra were measured by a JEOL JNM-ECS600 spectrometer at room temperature in deuterated dichloromethane and chloroform respectively with tetramethyl silane as the internal standard. (Due to the poor solubility of Cz-DICz in most solvents, the ^{13}C NMR spectrum could not be obtained). MALDI-TOF-MS data was performed on a Shimadzu AXIMA Performance MALDI-TOF instrument in positive detection modes. Elemental analyses were performed on a flash EA 1112 spectrometer.

Single-Crystal Structure. Diffraction data were collected on a Rigaku R-AXIS-RAPID diffractometer using the ω-scan mode with graphite-monochromator Mo•Kα radiation. The structure determination was solved with direct methods using the SHELXTL programs and refined with full-matrix least squares on F2. The corresponding CCDC reference number (2300406) and the data can be obtained free of charge from The Cambridge Crystallographic Data Centre via www.ccdc.cam.ac.uk/data_request/cif.

Computational methods. The calculations were performed using the Gaussian 16 package[S1], employing the density functional theory (DFT) and time-dependent density functional theory (TD-DFT) methods. The B3LYP and PBE0-D3BJ hybrid functionals were utilized.[S2-S5] The structures were optimized using DFT for S_0 state and TD-DFT for S_1 and T_1 states with a 6–31G* basis set. The solvent environment was simulated by the polarizable continuous model (PCM) with the integral equation formalism variant (IEFPCM), with toluene as solvent for all molecules. The MOMAP software package was used to calculate the absorption and emission spectra and analyze the vibrational modes in the emission spectrum.[S6-S9] The root-mean-square displacement (RMSD) between S0 and S1 structures was calculated using the VMD package.[S10] NTO analysis was performed using the Multiwfn package.[S11]

Photoluminescence Property Measurements. Organic films for optical measurements were fabricated by thermal evaporation under high vacuum onto clean quartz substrates. UV-vis absorption spectra were recorded by an Agilent 8453 spectrophotometer. Steady State

fluorescence spectra, fluorescence lifetime, and quantum efficiency were carried out with Edinburgh fluorescence spectrometer (FLS1000) with an integrating sphere. Transient spectra for prompt part were collected with a 365 nm picosecond pulsed LED (EPL365), while the delayed part was collected with a 312.8 nm pulse width tuning laser (VPL320).

Calculation Formulas for the Photophysical Parameters. The k_r and k_{RISC} rates were calculated to be 1.8×10^8 s^{-1} and 2.6×10^3 s^{-1}, respectively, using a previously reported method (*Adv. Mater.* **2018**, *30*, 1705406):

$k_p = 1/\tau_p$

$k_d = 1/\tau_d$

$k_{ISC} = k_P\phi_d/(\phi_d + \phi_p)$

$k_r = k_p\phi_p$

$k_{RISC} = k_d k_p \phi_d/(k_{ISC}\phi_p)$

where k_{ISC} is the intersystem crossing rate, k_{RISC} is the reverse intersystem crossing rate, k_r are the rate constants of the singlet radiative transition, k_p is the prompt decay rate, and k_d stands for the delayed decay rate, respectively. ϕ_p stands for the PLQY of the prompt part while ϕ_d for the delayed part.

Electrochemical measurements. The electrochemical properties of Cz-DICz were studied by cyclic voltammetry. As shown in Figure S6, the oxidation potentials calculated from the onset of the oxidation curves are -4.72 eV and -1.76 eV for Cz-DICz, vs. an Fc/Fc^+ standard, corresponding to the highest occupied molecular orbital (HOMO) and lowest unoccupied molecular orbital (LUMO) levels of -5.55 eV and -2.59 eV for Cz-DICz, using ferrocene as a reference.

Device fabrication and measurement of EL characteristics. All compounds were subjected to temperature-gradient sublimation under high vacuum before use. OLEDs were fabricated on the ITO-coated glass substrates with multiple organic layers sandwiched between the transparent bottom indium-tin-oxide (ITO) anode and the top metal cathode. Before device fabrication, the ITO glass substrates were pre-cleaned carefully. All material layers were deposited by vacuum evaporation in a vacuum chamber with a base pressure of 10^{-6} torr. The deposition system permits the fabrication of the complete device structure in a single vacuum pump-down without breaking vacuum. The deposition rate of organic layers was kept at 0.1 - 0.2 nm s^{-1}. The doping was conducted by co-evaporation from separate evaporation sources with different evaporation rates. The current density, voltage, luminance, external quantum efficiency, electroluminescent spectra and other characteristics were measured with a Keithley 2400 source meter and an absolute EQE measurement system in an integrating sphere at the

same time. The EQE measurement system is Hamamatsu C9920-12, which equipped with Hamamatsu PMA-12 Photonic multichannel analyzer C10027-02 whose longest detection wavelength is 1100 nm.

Synthetic procedures and characterization data.

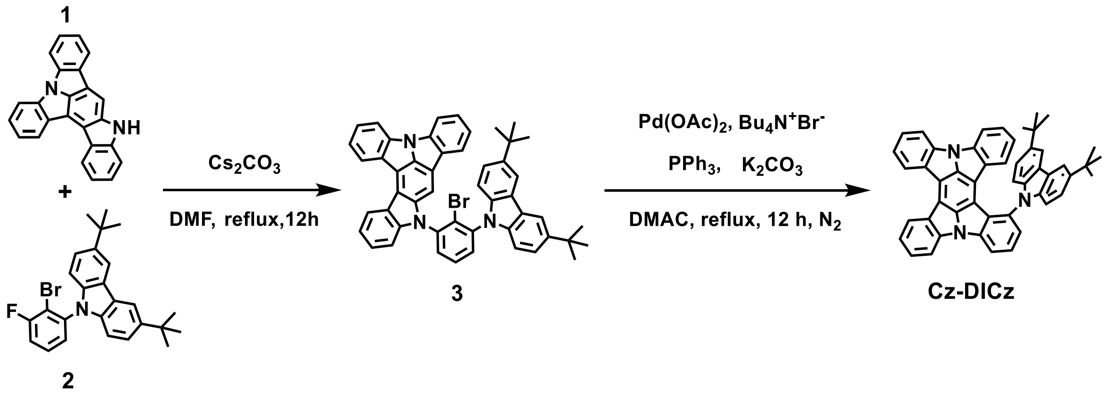

Scheme S1. Synthetic procedures of Cz-DICz.

Synthesis of 3: A mixture of **1** (2 g, 6.05 mmol)[S12], **2** (4.1 g, 9.08 mmol)[S13] and caesium carbonate (3.94 g, 12.1 mmol) were dissolved in N, N-dimethylformamide (60 mL). The mixture was stirred and refluxed for 12 h. After cooling to room temperature, the mixture was diluted with water and the crude product was recrystallized from hot ethanol to afford the product as a yellow powder (3.70g, Yield: 80%). ^{1}H NMR (600 MHz, Chloroform-*d*) δ 8.78 (d, *J* = 7.7 Hz, 1H), 8.71 (d, *J* = 7.8 Hz, 1H), 8.20- 8.16 (m, 3H), 8.03 (d, *J* = 8.0 Hz, 1H), 7.97 (d, *J* = 7.9 Hz, 1H), 7.82 (t, *J* = 3.2 Hz, 3H), 7.78-7.76 (m, 1H), 7.65-7.51 (m, 7H), 7.37 (t, *J* = 7.5 Hz, 1H), 7.32 (d, *J* = 8.1 Hz, 1H), 7.27 (d, *J* = 2.3 Hz, 1H), 7.18 (dd, *J* = 8.5, 2.3 Hz, 1H), 1.48 (t, *J* = 2.4 Hz, 18H). MALDI-TOF: (Calculated: 761.24, Found: 761.43).

Synthesis of Cz-DICz: A mixture of **3** (2 g, 2.62 mmol), tetrabutylammonium bromide (0.99 g, 2.62 mmol), K_2CO_3 (2.12 g, 13.1 mmol), triphenylphosphine (0.81 g, 2.62 mmol) and palladium (II) acetate (0.11 g, 0.40 mmol) in 30 mL N, N-dimethylacetamide (DMAC) was stirred and heated with reflux for 12 h under argon. Then it was cooled to room temperature and poured into ice-water (300 mL) The aqueous layer was separated and extracted with dichloromethane (100 mL). The combined organic layers were dried with magnesium sulphate, filtered, and evaporated under reduced pressure. The crude product Cz-DICz was purified by column chromatography (silica, petroleum ether/THF = 5:1) and recrystallized from CH_2Cl_2 and methanol as a yellow powder (0.94 g, yield: 53%). ^{1}H NMR (400 MHz, Chloroform-*d*) δ 8.53 (dd, *J* = 24.6, 7.7 Hz, 2H), 8.26 (d, *J* = 1.6 Hz, 2H), 8.18 (dd, *J* = 8.0, 2.0 Hz, 2H), 7.90 (d,

J = 8.0 Hz, 1H), 7.76 – 7.65 (m, 3H), 7.55 (q, J = 7.4 Hz, 2H), 7.43 (t, J = 7.5 Hz, 2H), 7.26 (d, J = 1.8 Hz, 1H), 7.24-7.13 (m, 4H), 6.39 (t, J = 7.4 Hz, 1H), 5.79 (d, J = 7.9 Hz, 1H), 1.41 (s, 18H). ^{13}C NMR could not be measured because of low solubility. MALDI-TOF: Calculated: 681.31, Found: 681.3225. Anal. Calcd (%) $C_{50}H_{39}N_3$: C, 88.07; H, 5.77; N, 6.16; Found: C, 88.95; H, 5.63; N, 6.42.

2. Other supplementary figures and tables

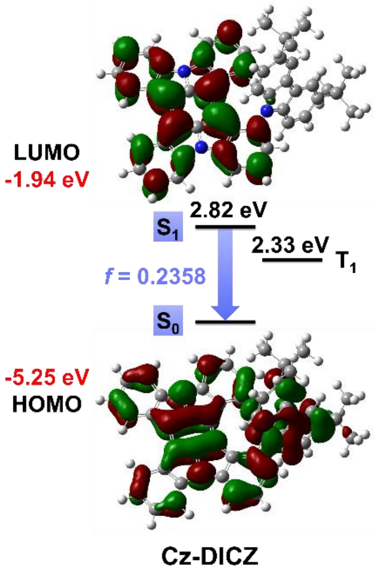

Figure S1. HOMO and LUMO distributions of Cz-DICz, calculated by the B3LYP/6-31G(d) method.

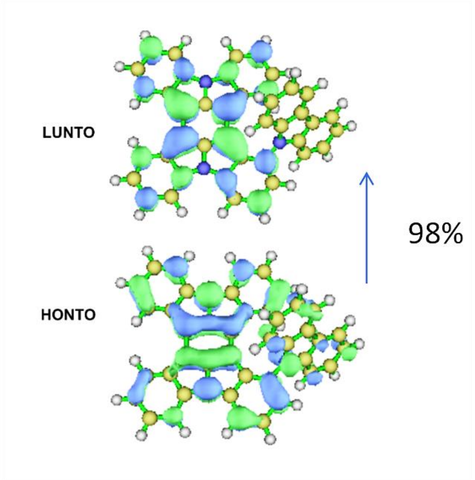

Figure S2. HONTO and LUNTO natural transition orbital distributions of Cz-DICz, calculated by the PBE0/6-31G(d) method.

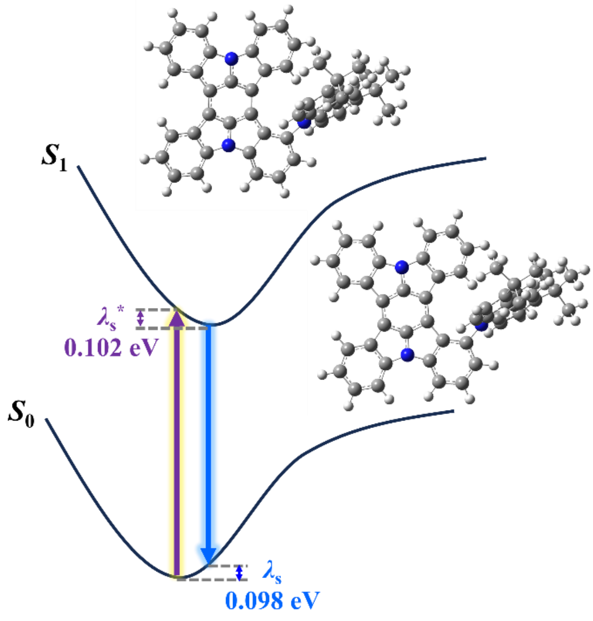

Figure S3. The optimized S_0 and S_1 structures of Cz-DICz and reorganization energies, calculated by the PBE0/6-31G(d) method.

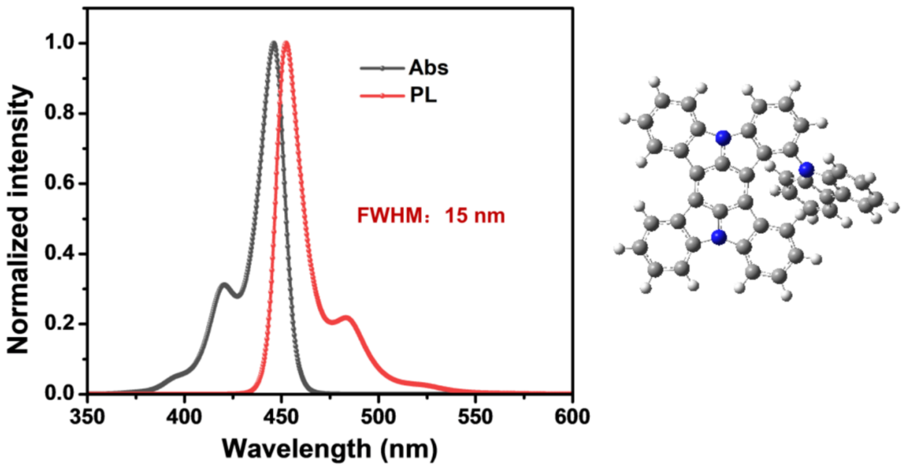

Figure S4. The absorption and emission spectra, calculated by the MOMAP package at the PBE0/6-31G(d) level. The frequency correction factor set to 0.9512.

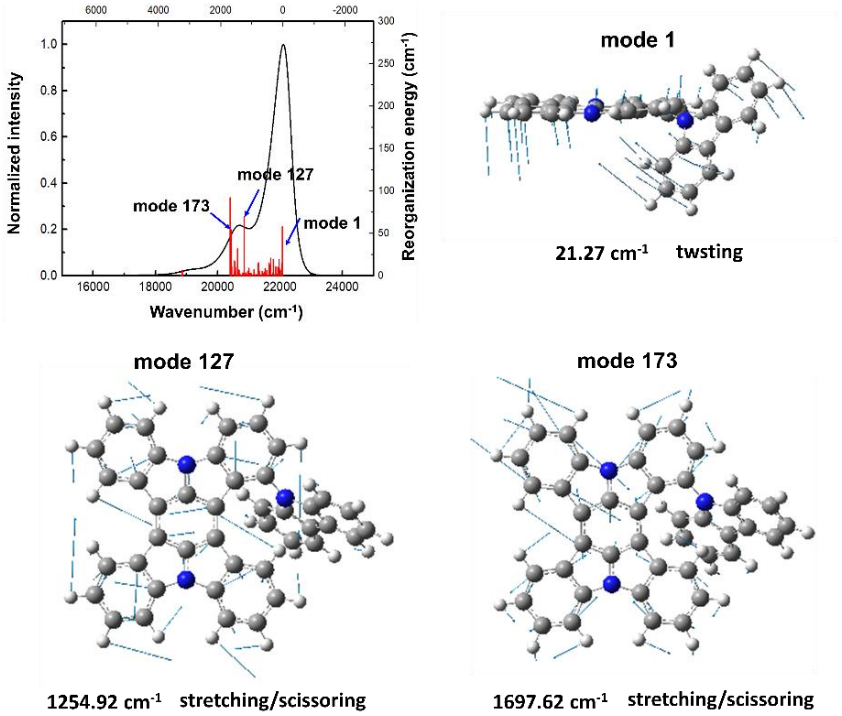

Figure S5. Vibrational modes in the S_0 state that contribute significantly to the emission spectrum of Cz-DICz, calculated by the PBE0/6-31G(d) method.

Table S1. Summary of TD-DFT calculations for Cz-DICz at the S_0 and S_1 structures at the B3LYP/6-31G(d) level.

Compound	Optimized Structure	Vertical	Wavelength (nm)	Energy (eV)	Oscillator Strength
Cz-DICz	S_0	$S_0 \rightarrow S_1$	439.28	2.8224	0.2358
		$S_0 \rightarrow T_1$	532.01	2.3305	0
	S_1	$S_1 \rightarrow S_0$	508.09	2.4405	0.1282
	T_1	$T_1 \rightarrow S_0$	579.11	2.1412	0

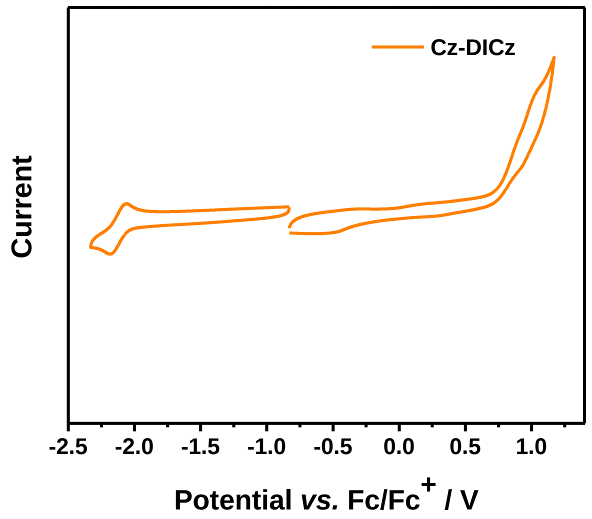

Figure S6. Cyclic voltammograms of Cz-DICz.

Table S2. Summary of the representative narrowband deep-blue MR emitters with emission wavelength around 460 nm.

Compound	Ref.	λ_{PL} (nm)	$FWHM_{PL}$ (nm)	λ_{EL} (nm)	$FWHM_{EL}$ (nm)
Cz-DICz	This work	457	14	460	18
DABNA-1	S14	460	30	459	28
t-DABNA	S15	457	26	465	27
t-DABNA-dtb	S15	465	22	473	23
t-DAB-DPA	S16,S17	456	23	459	26
4F-ν-DABNA	S18	457	14	464	18
4F-m-ν-DABNA	S18	455	14	461	18
BN1	S19	454	18	457	28
BN3	S19	456	17	458	23
BFCz-DABNA	S20	456	22	463	26
*p*BP-DABNA-Me	S21	462	22	464	23
BSBS-Z	S22	460	20	463	22
t3IDCz	S23	459	21	472	25
p3IDCz	S23	461	16	472	23

ɣ-Cb-B	S24	460	23	461	28
QAO	S25	466	32	468	39
tDAmDIPz	S26	457	17	459	18

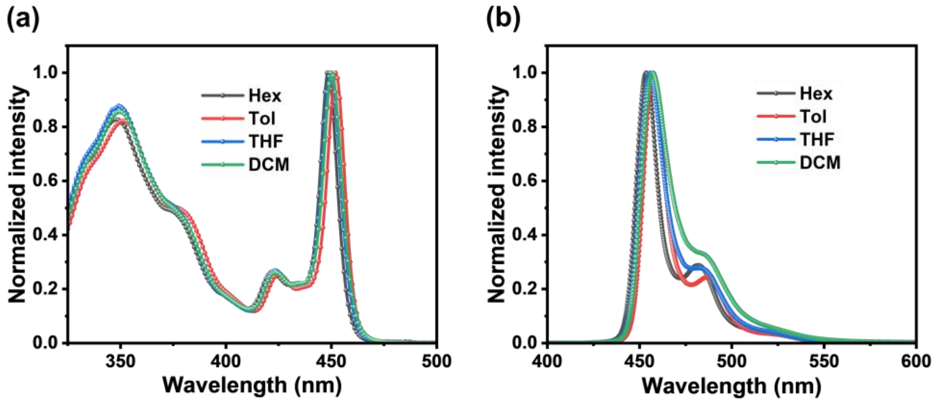

Figure S7. Normalized absoption (a) and fluorescent (b) emission spectra of Cz-DICz in different solvents.

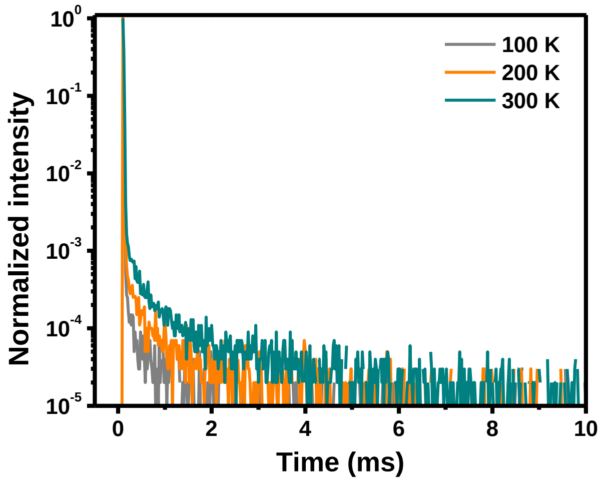

Figure S8. Transient decay spectra of mCBP:1 wt% Cz-DICz film at 100-300 K.

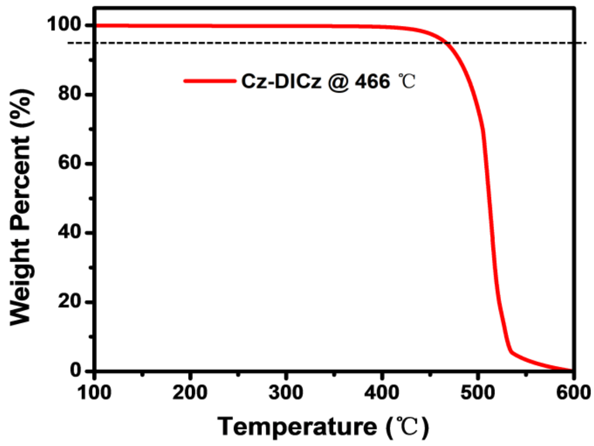

Figure S9. TGA thermogram of Cz-DICz.

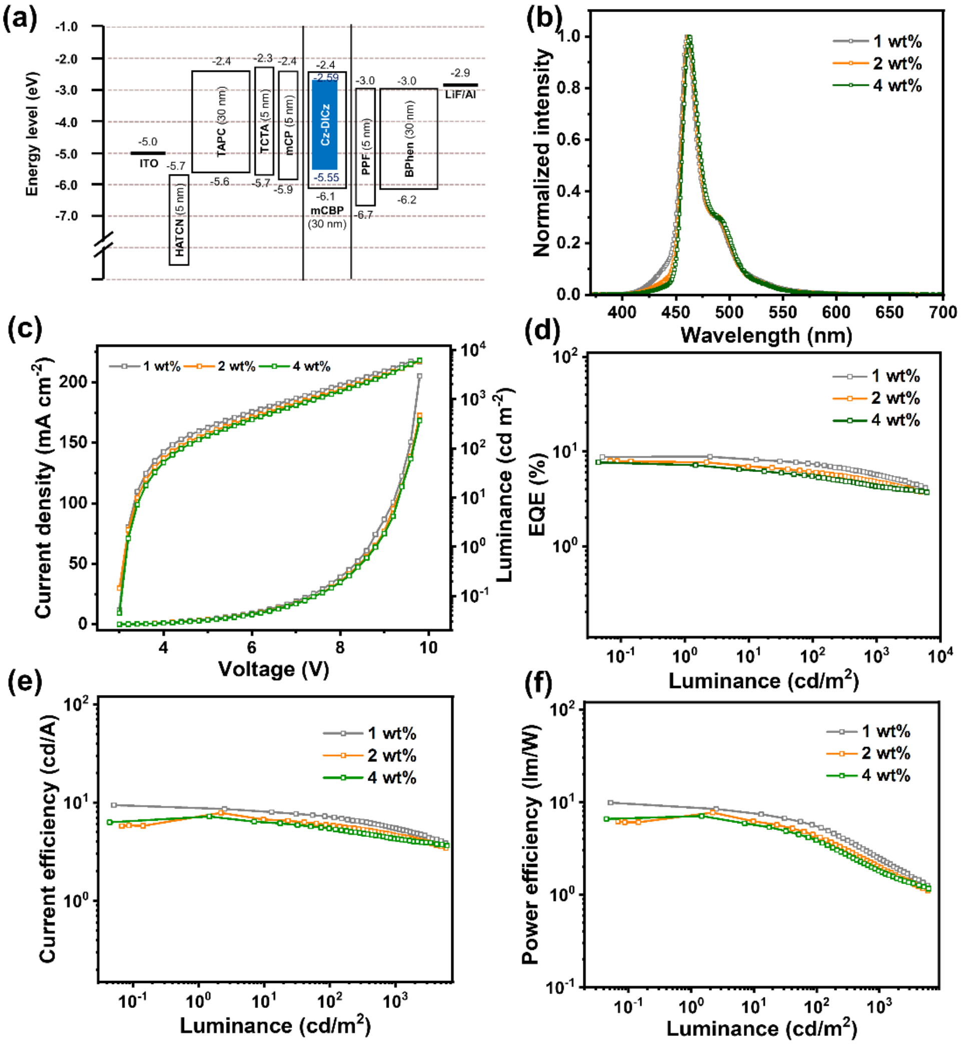

Figure S10. The performances of the non-sensitized devices. (a) The energy level diagram. (b) The EL spectra and CIE color coordinates at 10 mA/cm^2. (c) Current density-voltage-luminance characteristics. (d) The EQE_{max} values versus doping concentrations. (e) Current efficiency versus luminance curves. (f) Power efficiency versus luminance curves.

Table S3. Summary of the EL data of non-sensitized Cz-DICz devices.

Device	λ_{EL}[a)] [nm]	FWHM[b)] [nm]	V_{on}[c)] [V]	EQE[d)] [%]	CE[d)] [cd/A]	PE[d)] [lm/W]	CIE[e)] (x,y)
1 wt%	460	18	3.1	8.79/6.27	8.59/6.05	9.85/3.28	0.14,0.11
2 wt%	461	19	3.1	7.93/5.20	7.77/5.06	7.70/3.13	0.13,0.11
4 wt%	462	19	3.2	7.65/4.63	7.28/4.63	7.08/2.36	0.13,0.12

[a)] Maximum electroluminescence wavelength. [b)] Full width at half maximum of electroluminescence. [c)] Turn-on voltage when brightness is 0.2 cd m^{-2}. [d)] Maximum efficiency/ efficiency at 500 cd m^{-2}. [e)] Recorded at 10 mA/cm^2.

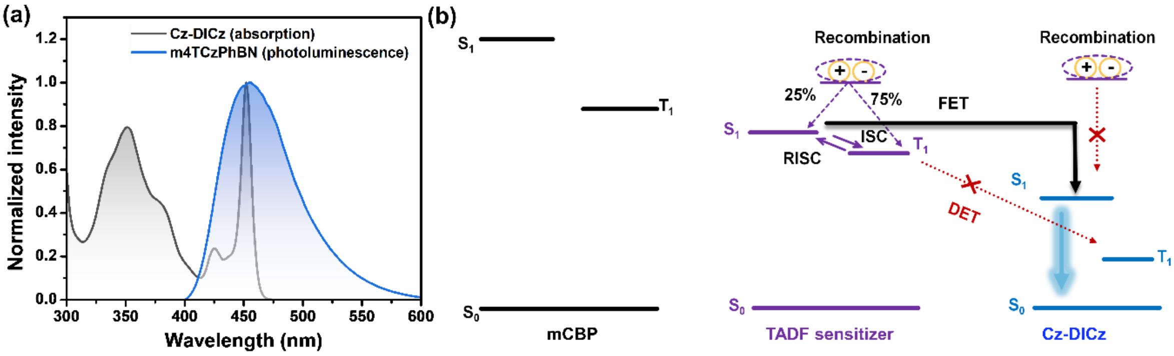

Figure S11. (a) The absorption of Cz-DICz and PL spectra of sensitizer m4TCzPhBN in the toluene solution (10^{-5} M). (b) The energy transfer process in TADF sensitized MR emitter (Förster: förster energy transfer; RISC: reverse intersystem crossing).

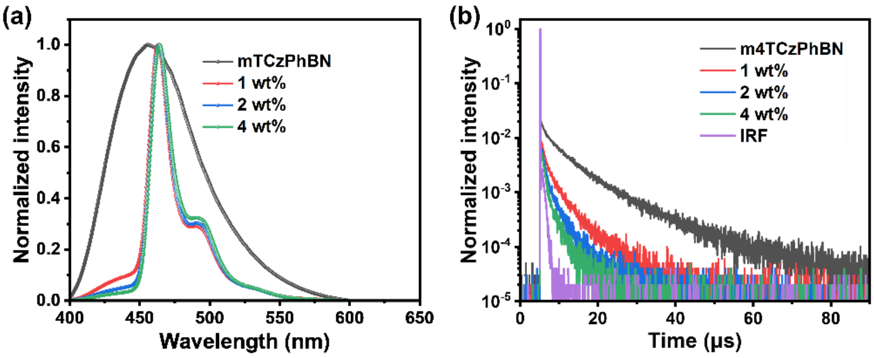

Figure S12. a) The normalized PL spectrum of mCBP:30 wt% m4TCzPhBN:1, 2, 4 wt% Cz-DICz films. b) Transient decay spectra of mCBP:30 wt% m4TCzPhBN:1, 2, 4 wt% Cz-DICz films at 300 K.

Table S4. Summarized photophysical properties of mCBP:30 wt% m4TCzPhBN:1, 2, 4 wt% Cz-DICz films.

	λ_{em} [nm]	FWHM [nm]	PLQY [%]	τ_p [ns]	τ_d [μs]
1 wt%	463	18	98.8	5.7	3.7
2 wt%	463	18	97.6	5.6	2.5
4 wt%	464	18	97.2	5.6	1.8

As shown in **Figure S12** and **Table S4**, both the TADF sensitized films exhibited similar deep-blue emissions at around 463 nm with an identical small FWHM of 18 nm, which was consistent with the PL results for the pristine ones. The fluorescence decays for the TADF sensitized films were fitted with double exponential expressions. The first short time scale decay (~5.6 ns) should be assigned to the prompt fluorescence of the Cz-DICz emitters, where similar exciton lifetimes could be observed in pristine films. The decay component (1.8-3.7 μs) can be ascribed to the Forster energy transfer from m4TCzPhBN to the Cz-DICz, which was much shorter than the pure MR emitters (~426 μs) and even shorter than m4TCzPhBN (~9.2 μs, *Adv. Mater*. **2020**, *32*, 1908355).

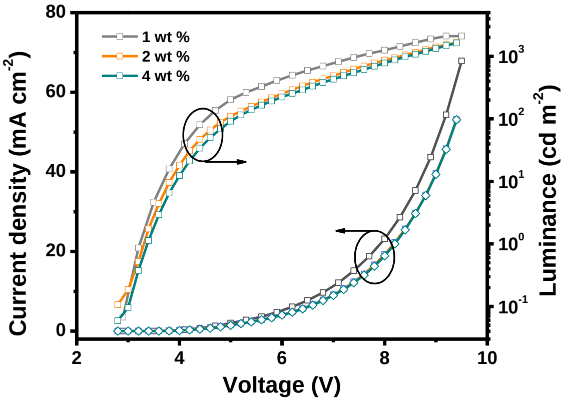

Figure S13. Current density-voltage-luminance characteristics of TADF sensitized Cz-DICz devices.

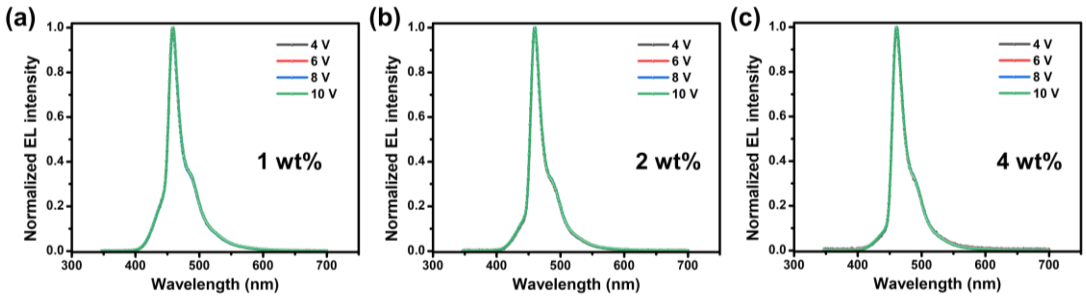

Figure S14. The EL spectra of TADF sensitized devices under different voltages.

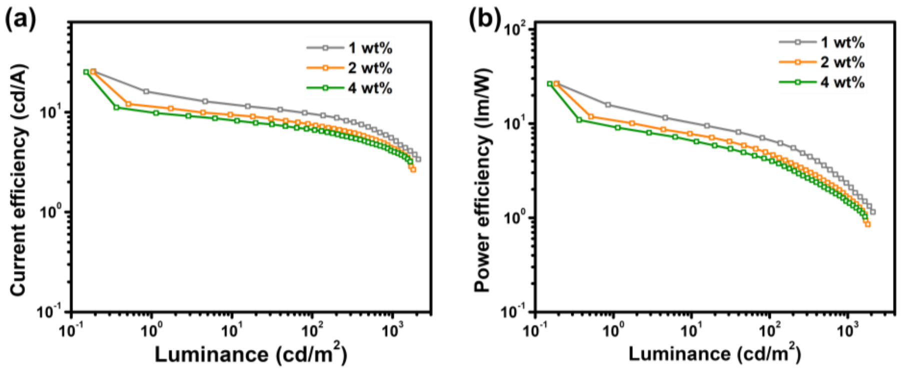

Figure S15. (a) Current efficiency versus luminance curves of the TADF sensitized devices. (b) Power efficiency versus luminance curves of the TADF sensitized devices.

Table S5. Summary of the EL data of TADF sensitized Cz-DICz devices.

Device	λ_{EL} [a)] [nm]	FWHM [b)] [nm]	V_{on} [c)] [V]	EQE [d)] [%]	CE [d)] [cd/A]	PE [d)] [lm/W]	CIE [e)] (x,y)
1 wt%	460	18	2.9	25.6/8.7	25.62/7.10	26.67/3.65	0.14,0.10
2 wt%	460	18	2.8	23.8/6.9	25.28/7.15	26.48/2.50	0.14,0.11
4 wt%	461	18	2.8	22.1/5.8	25.21/6.62	26.39/2.22	0.14,0.11

[a)] Maximum electroluminescence wavelength. [b)] Full width at half maximum of electroluminescence. [c)] Turn-on voltage when brightness is 0.2 cd m^{-2}. [d)] Maximum efficiency/ efficiency at 500 cd m^{-2}. [e)] Recorded at 10 mA/cm^2.

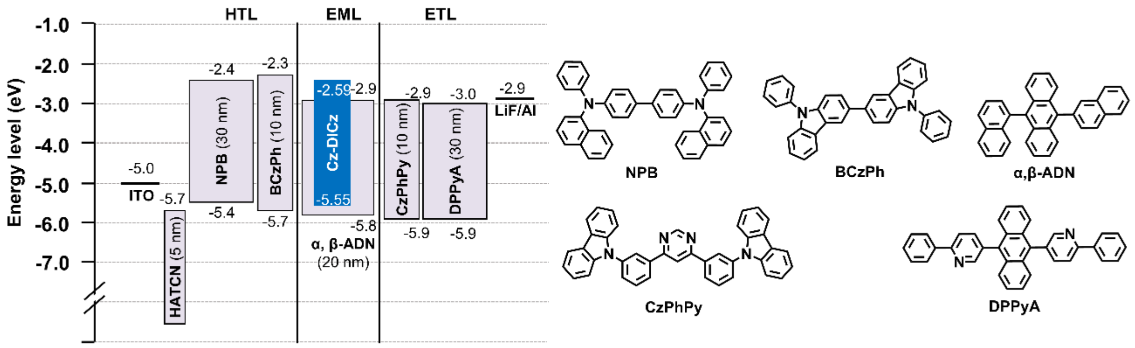

Figure S16. Energy level and molecular structure of the TTA device.

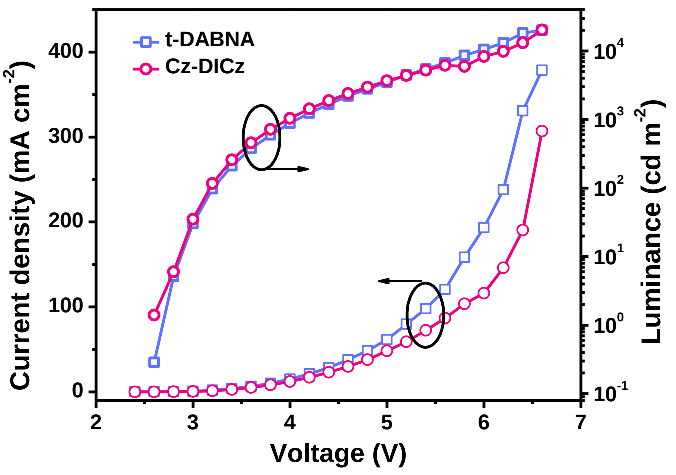

Figure S17. Current density-voltage-luminance characteristics of the TTA devices.

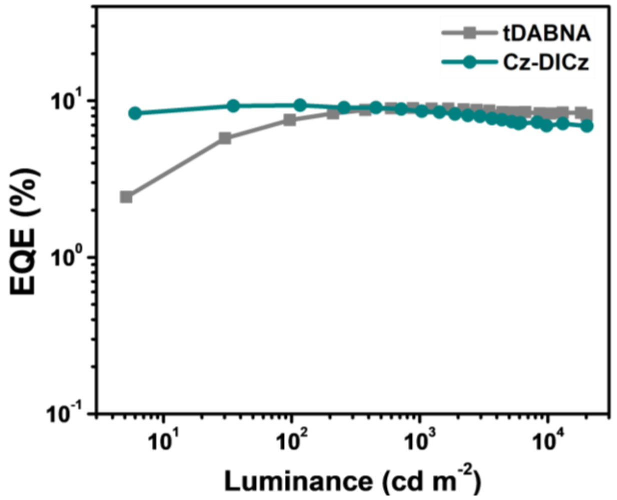

Figure S18. (a) External quantum efficiency versus luminance curves of the TTA devices.

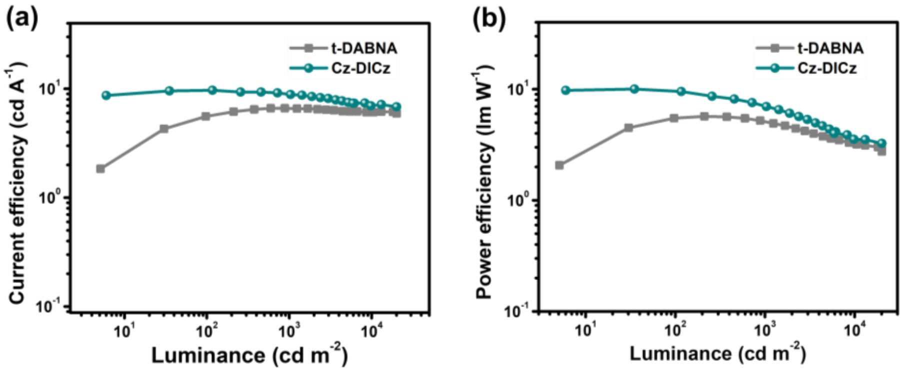

Figure S19. (a) Current efficiency versus luminance curves of the TTA devices. (b) Power efficiency versus luminance curves of the TTA devices.

Table S6. Summary of the EL data of the TTA devices.

Device	λ_{EL}[a)] [nm]	FWHM[b)] [nm]	$V_{turn-on}$[c)] [V]	EQE[d)] [%]	CE[d)] [cd/A]	CIE[e)] (x,y)
Cz-DICz	460	15	2.5	9.4/9.4/8.6	8.6/8.5/7.7	0.13,0.09
t-DABNA	459	25	2.6	9.0/7.5/9.0	6.6/5.5/6.4	0.14,0.09

[a)]Maximum electroluminescence wavelength. [b)]Full width at half maximum of electroluminescence. [c)]Turn-on voltage when brightness is 0.2 cd m^{-2}. [d)]Maximum efficiency/ efficiency at 100 cd m^{-2}/efficiency at 1000 cd m^{-2}. [e)]Recorded at 10 mA/cm^2.

Table S7. Crystal Data and Structure Refinement of Cz-DICz.

	Cz-DICz
empirical formula	C50 H39 N3
formula wt	681.84
crystal system	orthorhombic
T (K)	193
space group	P b c a
a/Å	9.1084(2)
b/Å	19.0622(5)
c/Å	42.0545(13)
α/ °	90
β/ °	90
γ/ °	90
V/Å3	7301.8(3)
Z	8
density, mg/m3	1.240
absorption coefficient, mm^{-1}	0.351
F (000)	2880.0
θ range/Å	3.658 to 57.022
no. of reflcns collected	7477
no. of unique reflns	4144
R(int)	0.1032
GOF	0.899
$R1$ [$I > 2\sigma(I)$]	0.0502

wR2 [$I > 2\sigma(I)$]	0.1271
R1 (all data)	0.1032
wR2 (all data)	0.1665

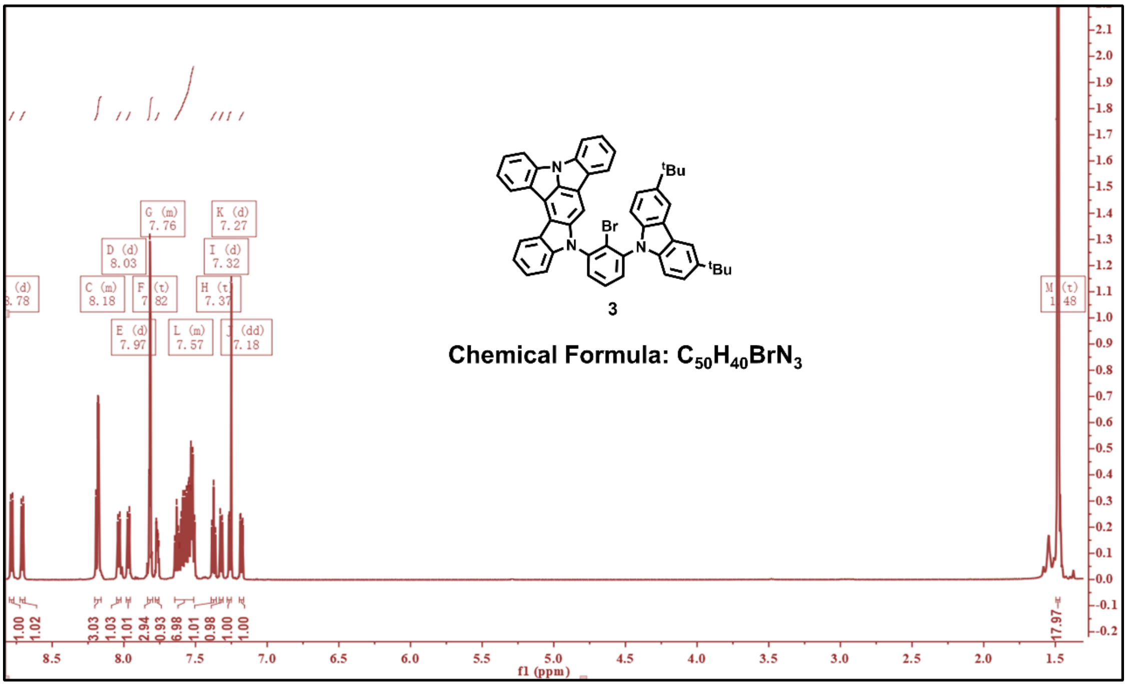

Figure S20. ^{1}H NMR spectrum of intermediate compound **3** in $CDCl_3$.

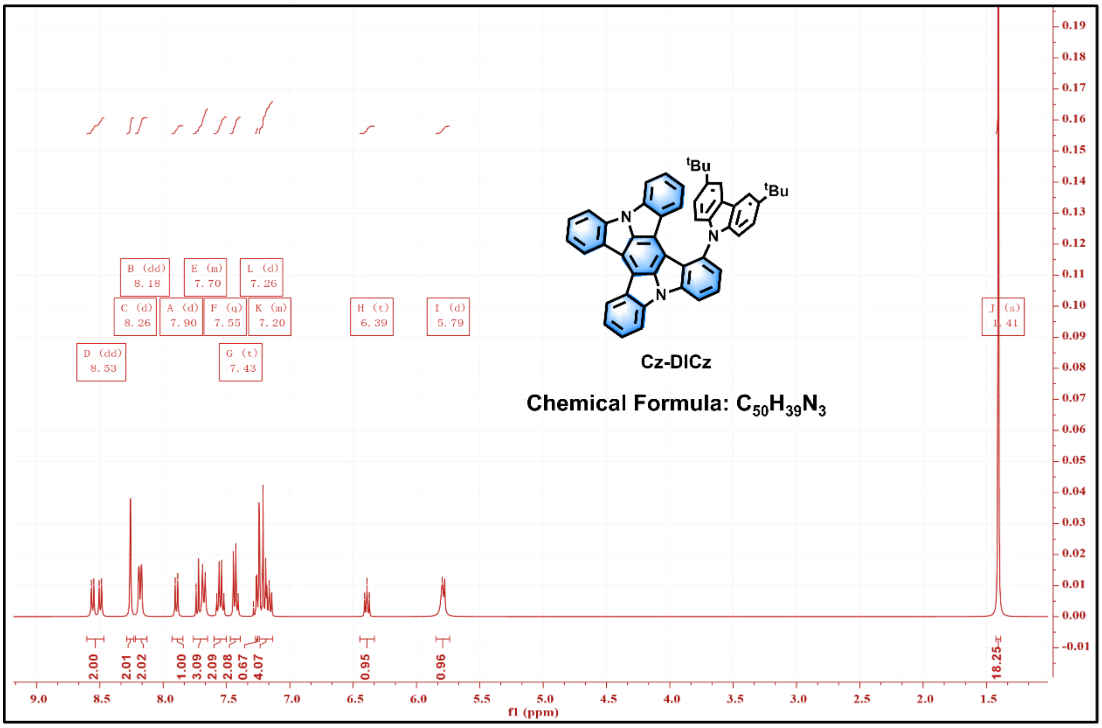

Figure S21. ^{1}H NMR spectrum of Cz-DICz in $CDCl_3$.

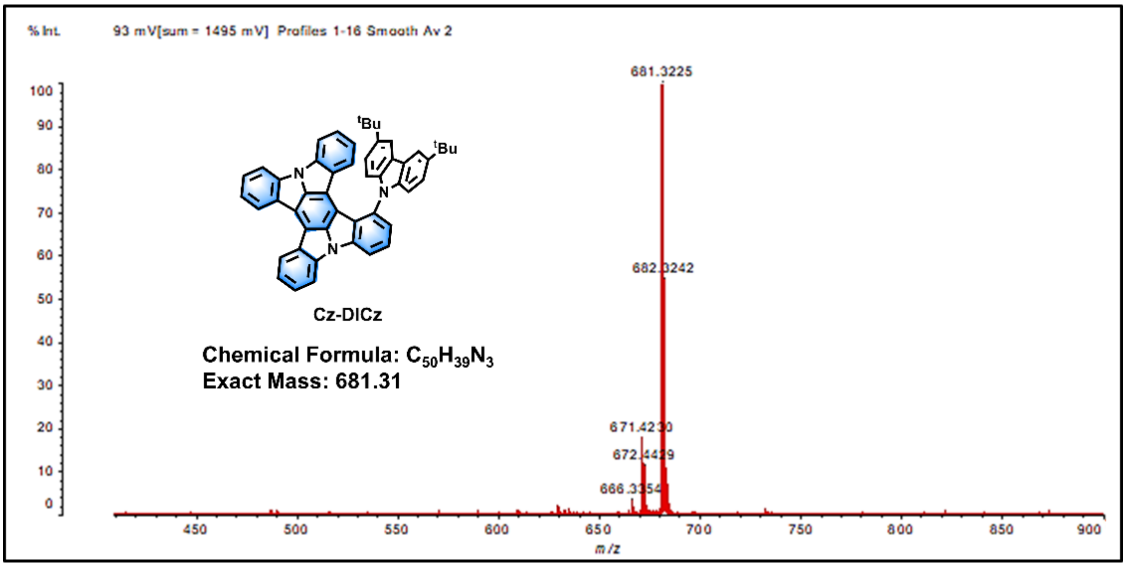

Figure S22. MALDI-TOF mass spectrum of Cz-DICz.

3. Reference

[S1] Gaussian 09, Revision A.02, M. J. Frisch, G. W. Trucks, H. B. Schlegel, G. E. Scuseria, M. A. Robb, J. R. Cheeseman, G. Scalmani, V. Barone, G. A. Petersson, H. Nakatsuji, X. Li, M. Caricato, A. Marenich, J. Bloino, B. G. Janesko, R. Gomperts, B. Mennucci, H. P. Hratchian, J. V. Ortiz, A. F. Izmaylov, J. L. Sonnenberg, D. Williams-Young, F. Ding, F. Lipparini, F. Egidi, J. Goings, B. Peng, A. Petrone, et al., *Gaussian, Inc.*, Wallingford, CT **2016**.

[S2] M. Ernzerhof, G.E. Scuseria, Assessment of the Perdew-Burke-Ernzerhof exchange-correlation functional, *J. Chem. Phys.* **1999**, 110, 5029–5036.

[S3] C. Adamo, V. Barone, Toward reliable density functional methods without adjustable parameters: The PBE0 model, *J. Chem. Phys.* **1999**, 110, 6158–6169.

[S4] J.P. Perdew, K. Burke, M. Ernzerhof, Generalized gradient approximation made simple, *Phys. Rev. Lett.* **1996**, 77, 3865–3868.

[S5] J.P. Perdew, K. Burke, M. Ernzerhof, Errata: Generalized gradient approximation made simple, *Phys. Rev. Lett.* **1997**, 78, 1396.

[S6] Peng, Q.; Niu, Y.; Shi, Q.; Gao, X.; Shuai, Z. CorrelationFunction Formalism for Triplet Excited State Decay: CombinedSpin−Orbit and Nonadiabatic Couplings. *J. Chem. Theory Comput.* **2013**, 9, 1132−1143.

[S7] Niu, Y.; Li, W.; Peng, Q.; Geng, H.; Yi, Y.; Wang, L.; Nan, G.;Wang, D.; Shuai, Z. MOlecular MAterials Property PredictionPackage (MOMAP) 1.0: a software package for predicting theluminescent properties and mobility of organic functional materials. *Mol. Phys.* **2018**, 116, 1078−1090.

[S8] Niu, Y.; Peng, Q.; Shuai, Z. Promoting-mode free formalism forexcited state radiationless decay process with Duschinsky rotationeffect.*Sci. China, Ser. B: Chem.* **2008**, 51, 1153−1158.

[S9] Peng, Q.; Yi, Y.; Shuai, Z.; Shao, J. Toward QuantitativePrediction of Molecular Fluorescence Quantum Efficiency: Role of Duschinsky Rotation. *J. Am. Chem. Soc.* **2007**, 129, 9333−9339.

[S10] Humphrey, W., Dalke, A. and Schulten, K., "VMD - Visual Molecular Dynamics" *J. Molec. Graphics.* **1996**, 14.1, 33-38.

[S11] Tian Lu, Feiwu Chen, Multiwfn: A Multifunctional Wavefunction Analyzer, *J. Comput. Chem.* **2012**, 33, 580-592.

[S12] Y. Zhang , J. Wei , L. Wang , T. Huang , G. Meng , X. Wang , X. Zeng , M. Du , T. Fan , C. Yin , D. Zhang and L. Duan , *Adv. Mater.* **2023**, *35*, 2209396.

[S13] M. Yang, I. S. Park, T. Yasuda, *J. Am. Chem. Soc.* **2020**, *142*, 19468-19472.

[S14] T. Hatakeyama, K. Shiren, K. Nakajima, S. Nomura, S. Nakatsuka, K. Kinoshita, J. Ni, Y. Ono, T. Ikuta, *Adv. Mater.* **2016**, *28*, 2777-2781.

[S15] J. Park, K.J. Kim, J. Lim, T. Kim, J.Y. Lee, *Adv. Mater.* **2022**, *34*, 2108581.

[S16] J. H. Kim, W. J. Chung, J. Kim, J. Y. Lee, *Mater. Today Energy* **2021**, *21*, 100792.

[S17] Y. Wang, Y. Duan, R. Guo, S. Ye, K. Di, W. Zhang, S. Zhuang, L. Wang, *Org. Electron.* **2021**, *97*, 106275.

[S18] K. Rayappa Naveen, H. Lee, R. Braveenth, K. Joon Yang, S. Jae Hwang, J. Hyuk Kwon, *Chem. Eng. J.* **2022**, *432*, 134381.

[S19] X. Lv, J. Miao, M. Liu, Q. Peng, C. Zhong, Y. Hu, X. Cao, H. Wu, Y. Yang, C. Zhou, J. Ma, Y. Zou, C. Yang, *Angew. Chem. Int. Ed.* **2022**, *61*, e202201588.

[S20] H. Lee, R. Braveenth, J. D. Park, C. Y. Jeon, H. S. Lee, J. H. Kwon, *ACS Appl. Mater. Interfaces* **2022**, *14*, 36927.

[S21] H. J. Cheon, S. J. Woo, S. H. Baek, J. H. Lee, Y. H. Kim, *Adv. Mater.* **2022**, *34*, 2207416.

[S22] I. S. Park, M. Yang, H. Shibata, N. Amanokura, T. Yasuda, *Adv. Mater.* **2021**, *31*, 2107951.

[S23] H. L. Lee, S. O. Jeon, I. Kim, S. C. Kim, J. Lim, J. Kim, S. Park, J. Chwae, W.-J. Son, H. Choi, J. Y. Lee, *Adv. Mater.* **2022**, *34*, 2202464.

[S24] H. Min, I. S. Park, T. Yasuda, *Angew. Chem. Int. Ed.* **2021**, *60*, 7643–7648.

[S25] Y. Yuan, X. Tang, X.-Y. Du, Y. Hu, Y.-J. Yu, Z.-Q. Jiang, L.-S. Liao, S.-T. Lee, *Adv. Opt. Mater.* **2019**, *7*, 1801536.

[S26] C. Cheng, Y. Zhu, T. Tsuboi, C. Deng, W. Lou, T. Liu, D. Wang, Q. Zhang, *Chem. Eng. J.* **2023**, *474*, 14569.
